# Real-World Effectiveness of a Booster Dose of the COVID-19 Vaccines among Japanese University Students

**DOI:** 10.3390/vaccines10081283

**Published:** 2022-08-09

**Authors:** Shunsuke Miyauchi, Toru Hiyama, Yukiko Nakano, Mahoko Yoshida, Atsuo Yoshino, Yoshie Miyake, Yuri Okamoto

**Affiliations:** 1 Health Service Center, Hiroshima University, 1-7-1 Kagamiyama, Higashihiroshima 739-8514, Japan; 2 Department of Cardiovascular Medicine, Graduate School of Biomedical and Health Sciences, Hiroshima University, 1-2-3 Kasumi, Minami-ku, Hiroshima 734-8551, Japan

**Keywords:** COVID-19, vaccine, booster dose, Omicron variant, close contact, university, vaccine hesitancy

## Abstract

With the spread of the Coronavirus disease 2019 (COVID-19), missing learning opportunities due to COVID-19 has been raised as a major concern for university education. We aimed to examine the effectiveness of a booster dose of COVID-19 vaccines among Japanese university students during the spread of the Omicron variant. We enrolled 249 students who became a close contact and 294 COVID-19-infected students though the Hiroshima University COVID-19 registration system. Infection rates of people in close contact with sick individuals and symptoms of infected students were examined. Close contacts who had received a booster dose showed a significantly lower infection rate (31%) compared with those with two doses (50%, *p* = 0.02) and the unvaccinated (71%, *p* = 0.002). Age- and sex-adjusted odds ratios of receipt of a booster dose vs. two doses and unvaccinated were 0.40 (95% confidence interval [CI], 0.23–0.70, *p* = 0.001) and 0.44 (95% CI, 0.25–0.77, *p* = 0.004), respectively. The incidence of severe fever (38.5°C or higher) was significantly less prevalent in those with a booster dose (16%) compared with two doses (40%, *p* = 0.002) and those who were unvaccinated (75%, *p* < 0.0001). Booster doses reduced infection rates among close-contact students and can help students to avoid missing learning opportunities.

## 1. Introduction

Coronavirus disease 2019 (COVID-19) caused by severe acute respiratory syndrome coronavirus 2 (SARS-CoV-2) was first reported in Wuhan, China, on 31 December 2019, and rapidly expanded worldwide [1]. On 24 November 2021, health authorities in South Africa reported a new SARS-CoV-2 variant, B.1.1.529 (Omicron) [2], and the World Health Organization (WHO) classified the Omicron variant as a variant of concern on 26 November 2021. With the spread of the Omicron variant, the effectiveness of COVID-19 vaccines has again become a controversial topic especially among the young population. A meta-analysis revealed that the effectiveness of two doses of a vaccine against infection and symptomatic disease of COVID-19 decreased approximately twenty-thirty percentage points by six months after the vaccination [3], and its effectiveness is limited against disease caused by the Omicron variant [4]. A booster dose of the COVID-19 vaccine was observed to increase protection against the Omicron variant [4], and speeding up of the coverage of booster dose vaccinations is recommended given the prevalence of the Omicron variant [5]. Missing learning opportunities due to COVID-19 has been raised as a major concern for university education, and it is hypothesized that a booster dose may be able to play a key role in preventing students from education losses. However, young populations tend to have lower acceptance of COVID-19 vaccines, which is described as “vaccine hesitancy” [6,7]. Vaccine hesitancy is also observed among university students including students in health professions [8,9]. To tackle this issue, accumulation of real-world evidence related to the effectiveness of COVID-19 vaccine in young populations remains an urgent challenge.

This study aimed to evaluate the real-world effectiveness of a booster dose of COVID-19 vaccines among Japanese university students using the Hiroshima University COVID-19 registration system.

## 2. Materials and Methods

### 2.1. Enrollment

**Figure 1:**
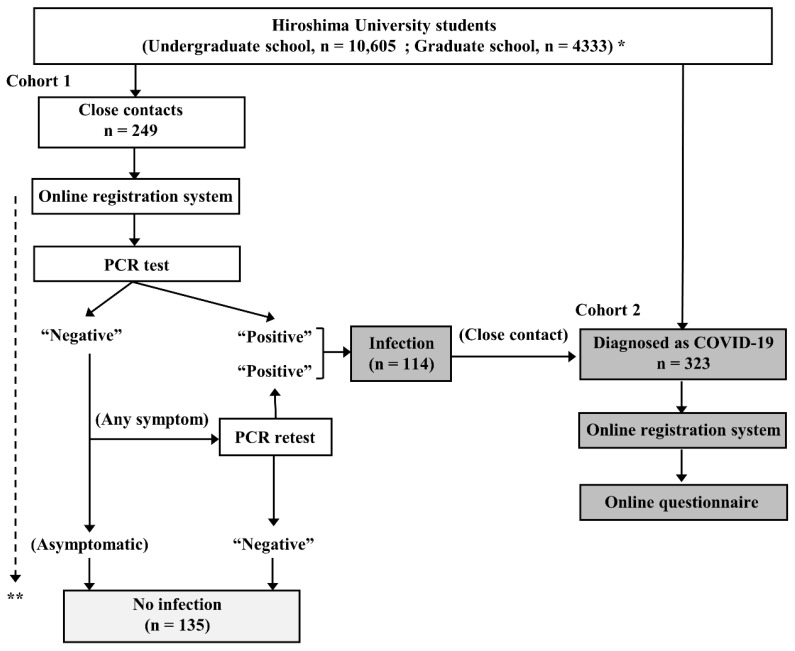
Study chart. * As of January 1, 2022. ** Close contacts were followed up for at least 10 days from their last contact with a COVID-19 patient. COVID-19 = coronavirus disease 2019; PCR = polymerase chain reaction.

This observational study was conducted at Hiroshima University, Higashihiroshima and Hiroshima, Japan, from 1 January to 30 April 2022. The study design is depicted in Figure 1. Students at Hiroshima University who were diagnosed as having COVID-19 or who became a close contact of a COVID-19 patient were firmly instructed to register their status with the online COVID-19 registration system of Hiroshima University and fill out the associated questionnaire.

In cohort 1, 249 consecutive students who had become a close contact of a COVID-19 patient during the study period were prospectively enrolled. All of these students underwent at least one polymerase chain reaction (PCR) test for SARS-CoV-2 even if they were asymptomatic according to the instruction of the public health service. The students with a negative result at the first PCR test were followed up for at least 10 days from their last contact with a COVID-19 patient by public health services. If a new onset of symptoms occurred during the follow-up period, they underwent PCR retesting. The students with a positive result at least one PCR test (n = 114) were also enrolled in cohort 2.

In cohort 2, 323 consecutive students who had been diagnosed as having COVID-19 during the study period were prospectively enrolled. Of the 323 students, 114 fulfilled the criterial of close contact and they were redundantly enrolled in cohort 1. Information was collected from each student concerning their sex, age, type and doses of COVID-19 vaccine received, and symptoms, including fever, cough, pharyngeal pain, headache, muscle and joint pain, dysgeusia, dyssomnia, nausea, diarrhea, and chest pain by an online questionnaire. Fever was defined as an axillary temperature of 37.5 °C or higher. Other symptoms were dependent on the students’ self-assessments. Of the 323 students, 29 did not fulfilled the questionnaire and were excluded. The other 294 were finally included in the analysis.

This study was approved by the Ethical Committee of Hiroshima University (approval number: E–143–3). Informed consent was waived by the Institutional Review Board due to the observational nature of the study and due to the fact that participant identifiers were completely encrypted before analysis.

### 2.2. Definition of a Close Contact

A close contact fulfilled the following four criteria: (a) contact with a COVID-19 patient between 2 days before and 14 days after the onset of symptoms, (b) no use of masks, (c) distance of less than 1 m, and (d) more than 15 min of contact [10]. Direct physical contact during physical education and club activities was also considered a close contact. The determination of a close contact was based on both the information of the students’ self-reporting on the online questionnaire and on an interview report provided by the public health service or Hiroshima University Health Service Center.

### 2.3. COVID-19 Vaccination

In Japan, a third-dose vaccination with either a BNT162b2 vaccine or a half dose (50 μg) of an mRNA-1273 vaccine was introduced to all people aged 12 years or older who had received a second vaccination dose at least 6 months before from 1 December 2021 [11]. Vaccination was not mandatory for students at Hiroshima University, and the decision to get vaccinated was left to the students’ intent. Information on a COVID-19 vaccine received, including any three doses of a BNT162b2 (Comirnaty, Pfizer–BioNTech, Mainz, Germany/New York, NY, USA) or mRNA-1273 (Spikevax, Moderna, Cambridge, MA, USA) vaccine (including mixed-product regimens) or any two doses of a BNT162b2 or mRNA-1273 vaccine, and being unvaccinated was collected based on the students’ self-reporting. Three students in cohort 1 and 2 students in cohort 2 had received one dose of vaccine, and those cases were counted as unvaccinated. Mass vaccinations for students were conducted at Hiroshima University using mRNA-1273 vaccine (first dose, from 21 June to 2 July 2021; second dose, from 26 July to 2 August 2021; third dose, from 1–7 and 15–16 March 2022). Thus, most vaccinated students received an mRNA-1273 vaccine during those periods. Although the ChAdOx1 nCoV-19 vaccine (Vaxzevria, AstraZeneca, Oxford, UK) was approved in May 2021, it had not yet been deployed on a large scale in Japan at the time of the study, and no enrolled student received the ChAdOx1 nCoV-19 vaccine.

### 2.4. Statistical Analysis

Continuous data (age) are presented as means ± standard deviations. Categorical variables (sex, transmission, and symptoms) are presented as percentages of the group total. Multiple comparisons of the categorical variables (incidence of transmission and symptoms) between the three groups of vaccine doses (three doses, two doses, and unvaccinated) were performed by Fisher’s exact test followed by Steel-Dwass post hoc test. The odds ratio (OR) was used as an estimate of the effectiveness of three doses of vaccine compared with two doses and unvaccinated and was calculated using univariate and multivariable logistic regression analyses. The multivariate model included age and sex. *p* Values of <0.05 indicated statistical significance. JMP software version 15.0 (SAS Institute, Cary, NC, USA) was used to perform all statistical analyses.

## 3. Results

### 3.1. Cohort 1: Vaccine Doses and Infection Rates among Close Contacts

During the study period, 249 students were enrolled as close contacts (cohort 1). Their mean age was 21.5 ± 2.7 (years), and 158 (63%) were male. A total of 83 (33%) students received 3 vaccination doses and 145 (58%) received 2 doses, whereas 21 (9%) were unvaccinated. No students in cohort 1 had been diagnosed as COVID-19 before enrollment.

**Figure 2:**
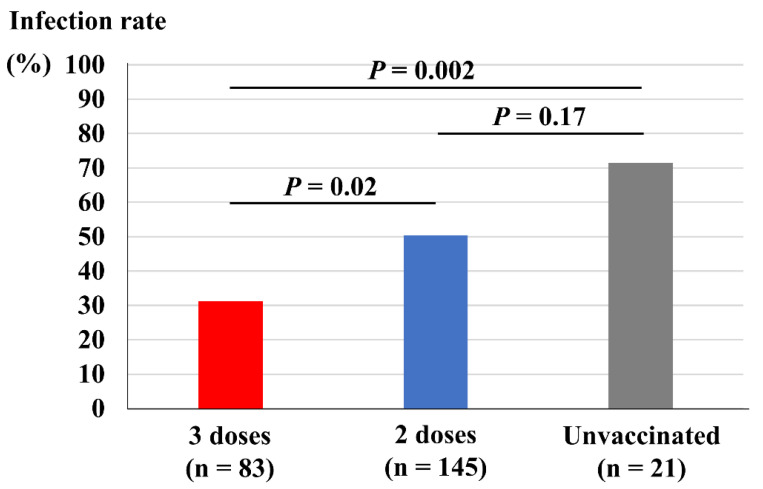
Vaccine doses and infection rate among the close contacts.

Figure 2 depicts comparisons of vaccine dose and infection rate among the close contacts. Close contacts who had received three doses of vaccine showed a significantly lower infection rate (31%) compared both with those who had received two doses of vaccine (50%, *p* = 0.02) and those who remained unvaccinated (71%, *p* = 0.002). However, those who had received two doses of vaccine did not show a significant reduction in infection rate compared with those who remained unvaccinated (*p* = 0.17).

Table 1 shows the ORs of infection after three doses of vaccine compared with two doses and unvaccinated. Unadjusted and age- and sex-adjusted ORs of three doses vs. two doses and unvaccinated were 0.40 and 0.44, respectively. Unadjusted and age- and sex-adjusted ORs of three doses vs. unvaccinated were 0.18 and 0.18, respectively. Unadjusted and age- and sex-adjusted ORs of three doses vs. two doses were 0.45 and 0.49, respectively.

### 3.2. Cohort 2: Vaccine Doses and Symptoms among COVID-19-Infected Students

During the study period, 323 students were diagnosed as having COVID-19 and were enrolled in cohort 2. A total of 294 students who fulfilled the online questionnaire were included in the analyses. Their mean age was 21.7 ± 3.4 (years), and 198 (67%) were male. A total of 63 (21%) students received 3 vaccination doses, 196 (67%) received 2 vaccination doses, and 34 (12%) were unvaccinated. No students in cohort 2 had been diagnosed as COVID-19 before enrollment.

**Figure 3:**
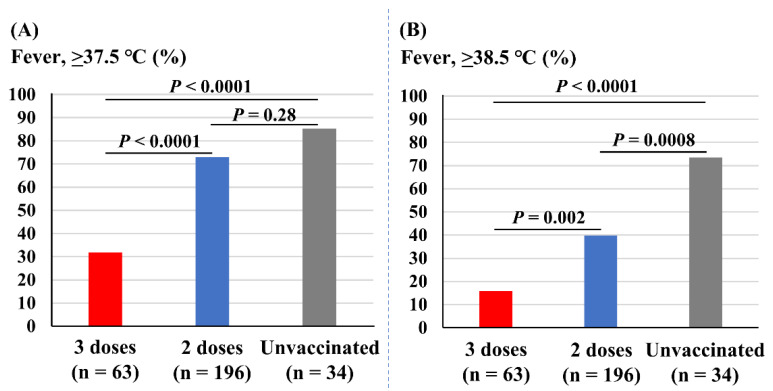
Associations of vaccine doses and incidences of fever. (**A**) Incidence of fever (37.5 °C or higher). (**B**) Incidence of fever of 38.5 °C or higher.

Figure 3 depicts the association of vaccine doses with incidences of fever. The incidence of fever (37.5 °C or higher) was significantly less prevalent in those receiving three doses of vaccine (32%) compared with those receiving two doses (73%, *p* < 0.0001) and the unvaccinated (85%, *p* < 0.0001). The incidence of fever was not different between those receiving two doses and the unvaccinated students (*p* = 0.28; Figure 3A). The incidence of severe fever (38.5 °C or higher) was also significantly less prevalent in those with three doses of vaccine (16%) compared with two doses (40%, *p* = 0.002) and the unvaccinated (75%, *p* < 0.0001). In addition, those with two doses of vaccine experienced fewer incidences of fever of 38.5 °C or higher compared with the unvaccinated students (*p* = 0.0008; Figure 3B).

Table 2 reveals the ORs of fever (37.5 °C or higher and 38.5 °C or higher) of those receiving three doses of vaccine compared with two doses and the unvaccinated students. Unadjusted and age- and sex-adjusted ORs of fever (37.5 °C or higher) after three doses vs. two doses and the unvaccinated were 0.16 and 0.16, respectively. Unadjusted and age- and sex-adjusted ORs after three doses vs. two doses were 0.17 and 0.17, respectively. Unadjusted and age- and sex-adjusted ORs after three doses vs. those who were unvaccinated were 0.09and 0.08, respectively. In contrast, unadjusted and age- and sex-adjusted ORs of fever (38.5 °C or higher) after three doses vs. two doses and unvaccinated were 0.23 and 0.20, respectively. Unadjusted and age- and sex-adjusted ORs after three doses vs. two doses were 0.28 and 0.25, respectively. Unadjusted and age- and sex-adjusted ORs after three doses vs. those who were unvaccinated were 0.06 and 0.06, respectively.

**Figure 4:**
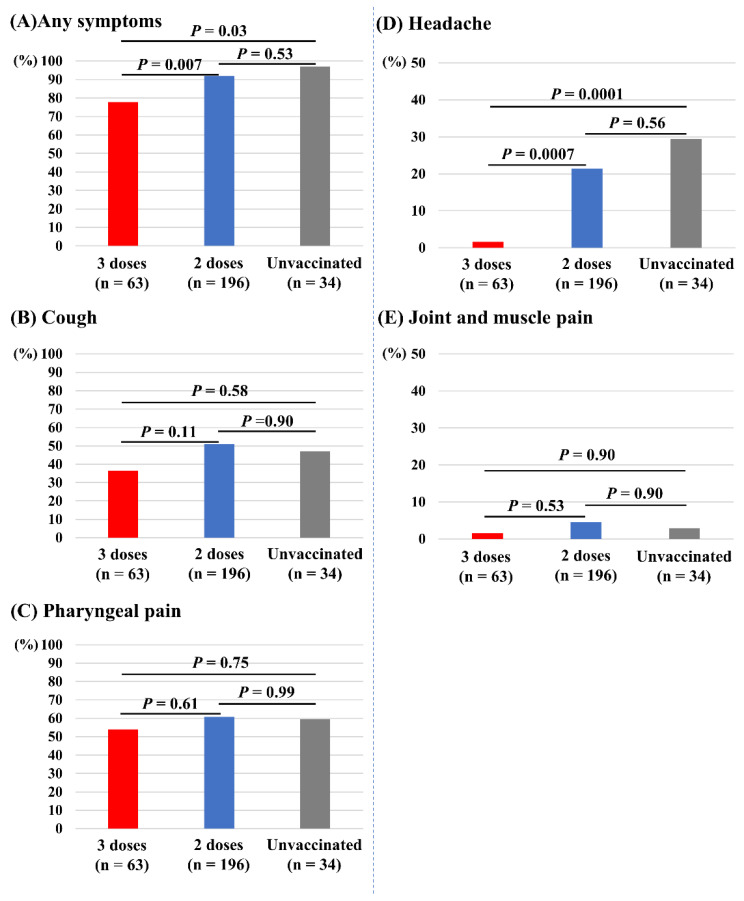
Associations of vaccine doses and symptoms other than fever. (**A**) Any symptoms. (**B**) Cough. (**C**) Pharyngeal pain. (**D**) Headache. (**E**) Joint and muscle pain.

Figure 4 depicts the associations of vaccine doses and other symptoms. Those who received three doses of vaccine had significantly fewer incidences of any symptoms (78%) compared with those receiving two doses (92%, *p* = 0.007) and the unvaccinated (97%, *p* = 0.03; Figure 4A). Those who received three doses of vaccine also had significantly fewer incidences of headache (2%) compared with those receiving two doses (21%, *p* = 0.0007) and the unvaccinated (29%, *p* = 0.0001; Figure 4D). The incidences of cough (Figure 4B), pharyngeal pain (Figure 4C), and joint and muscle pain (Figure 4E) did not differ between the three groups. Dysgeusia and dyssomnia (n = 7), nausea (n =2), diarrhea (n = 3), and chest pain (n = 1) were less common in the overall study population.

## 4. Discussion

The major findings of this study were as follows: three doses (booster dose) of vaccine were associated with (a) a lower infection rate among COVID-19 close contacts compared with both two doses and no vaccination; (b) lower incidences of fever (≥37.5 °C and ≥38.5 °C) among COVID-19-infected students compared with both two doses and no vaccination; and (c) lower incidences of symptoms and headache among COVID-19-infected students compared with both two doses and no vaccination.

After initial reports indicated that the booster dose was effective in reducing the rates of COVID-19 infection and severe disease against the B.1.617.2 (delta) variant in the elderly population [12], the effectiveness of the booster dose was demonstrated to apply to every age group including teens and those in their twenties [13]. A large-scale observational study demonstrated the effectiveness of booster dose compared with only two doses on preventing both transmission and becoming severe [14]. Since then, the Omicron variant classified as a variant of concern by WHO in November 2021 has spread worldwide, initiating replacement of the Delta variant within only one month [5]. In Japan, the Omicron variant was expected to invade at the end of December 2021, and it subsequently caused the largest increase in COVID-19 cases ever, the so-called “6th wave”, from January 2022 [15]. The Omicron variant has high transmissibility reported to be 3.2 times higher than that of the Delta variant, and its doubling time is approximately three days [16]. Although still under debate, the latest evidence indicates the effectiveness of a booster dose of COVID-19 vaccines against the Omicron variant. Furukawa et al. reported that booster vaccination with the BNT162b2 mRNA vaccine was associated with induction of a high level of neutralizing antibodies against the Omicron variant, whereas two doses did not induce sufficient neutralizing antibodies [17]. Similar results have also been observed in some of the latest reports [14,18]. However, real-word evidence that reveals the effectiveness of a booster dose in reducing risks of COVID-19 transmission and severe disease during the Omicron-dominant spread is limited (especially for young populations).

Although the emergence of the Omicron variant has sped up the recommendation for vaccination with a booster dose and the COVID-19 vaccines have been globally available, the success of vaccine coverage depends heavily on the vaccine acceptability of individuals. Vaccine hesitancy has been identified as a significant barrier to overcoming the COVID-19 pandemic [6,19]. Previous reports revealed that the prevalence of COVID-19 vaccine hesitancy is higher among the young population [6,20]. A study from the United States reported that suspicions about the efficacy and effectiveness of COVID-19 vaccines is one of the significant factors related to vaccine hesitancy among medical students [9]. Providing real-world evidence of the effectiveness of COVID-19 vaccines may help to tackle this issue of vaccine hesitancy among young populations.

University students have many opportunities to become a close contact through attendance at classes, clubs, and extracurricular activities, and dining with a number of other students. At the present time (May 2022) in Japan, close contacts are required to self-isolate for at least 7 days, and close contacts with disease transmission are required to undergo at least 10 days of self-isolation. Such self-isolation could have considerable negative impacts on the education and mental health of those affected [21], and a significant concern of university staffs, students, and their parents is the risk of missed learning and working (in addition to the risk of transmission) [22]. The present study clarified that a booster dose of the COVID-19 vaccines contributes to a lower transmission rate among close contacts with a OR of 0.40 compared with two doses and no vaccination. This indicates that a booster dose can potentially minimize the self-isolation period by avoiding transmission. Currently, shortening or even abolishing the self-isolation period of close contacts is being discussed, and once the regulations have been relaxed, school life will return to normal, although the spread of COVID-19 is still not going to be controlled. A booster dose of a COVID-19 vaccine can help to reduce the risk of transmission even in close-contact situations and thus may minimalize the self-isolation period and related educational losses. In addition, a significant number of people recovering from COVID-19 are still experiencing related symptoms for several months, including fatigue, myalgias, headaches, and cardiorespiratory and gastrointestinal symptoms labeled as “post COVID syndrome” [23]. Post COVID syndromes are also observed in young adults who do not have any underlying illnesses [24] and may have a negative impact on their learning after recovery. This study showed that a booster dose was associated with a lower incidence and severity of acute symptoms related to COVID-19 infection. Further study is expected to clarify the positive impact of a booster dose of vaccine on preventing physical and mental symptoms after COVID.

This study provides real-world evidence of the positive impact of a booster dose of COVID-19 vaccine for Japanese university students during the spread of the Omicron variant. The present findings may help to eliminate students’ suspicions about the effectiveness of COVID-19 vaccines and provide an opportunity for those who have not yet accepted COVID-19 vaccines to reconsider vaccination.

### Study Limitations

The present study has several limitations. First, although students who had become a close contact with infected person or who had become infected themselves were firmly instructed to register with the online system, some may not have done so, and these students may have been missed. Second, we did not examine the responsible variant in each case. However, most cases were estimated to be infected by the Omicron variant during the study period [15]. Third, we did not have information on vaccination dates and duration after the vaccination. Fourth, information on symptoms was based on the students’ self-reports and not confirmed by medical professionals. Finally, the sample size is small.

## 5. Conclusions

We revealed the effectiveness of a booster dose of COVID-19 vaccines among Japanese university students. Booster doses reduce the transmission rate of close contacts and can help students to avoid missing learning opportunities.

## Figures and Tables

**Table 1:** Odds ratios of infection after three doses of vaccine compared with two doses and those who were unvaccinated.

	Univariate		Multivariate *	
	OR (95% CI)	*p* Value	OR (95% CI)	*p* Value
Three doses vs. two doses and unvaccinated	0.40 (0.23−0.70)	0.001	0.44 (0.25−0.77)	0.004
Three doses vs. two doses	0.45 (0.26−0.79)	0.006	0.49 (0.27−0.88)	0.02
Three doses vs. unvaccinated	0.18 (0.06−0.52)	0.002	0.18 (0.06−0.54)	0.002

* Multivariate model including age and sex. OR, odds ratio; CI, confidence interval.

**Table 2:** Odds ratios of fever of ≥37.5 °C or ≥38.5 °C after three doses of vaccine compared with two doses and those who were unvaccinated.

	Univariate		Multivariate *	
	OR (95% CI)	*p* Value	OR (95% CI)	*p* Value
**Fever, 37.5 °C or higher**				
Three doses vs. two doses and unvaccinated	0.16 (0.09−0.29)	<0.0001	0.16 (0.09−0.29)	<0.0001
Three doses vs. two doses	0.17 (0.09−0.32)	<0.0001	0.17 (0.09−0.32)	<0.0001
Three doses vs. unvaccinated	0.09 (0.03−0.26)	<0.0001	0.08 (0.03−0.25)	<0.0001
**Fever, 38.5 °C or higher**				
Three doses vs. two doses and unvaccinated	0.23 (0.11−0.47)	<0.0001	0.20 (0.10−0.43)	<0.0001
Three doses vs. two doses	0.28 (0.13−0.58)	0.0007	0.25 (0.12−0.53)	0.0004
Three doses vs. unvaccinated	0.06 (0.02−0.18)	<0.0001	0.06 (0.02−0.17)	<0.0001

* Multivariate model including age and sex. OR, odds ratio; CI, confidence interval.

## Data Availability

The data presented in this study are available on request from the corresponding author.
